# The feasibility investigation of AI -assisted compressed sensing in kidney MR imaging: an ultra-fast T2WI imaging technology

**DOI:** 10.1186/s12880-022-00842-1

**Published:** 2022-07-04

**Authors:** Yanjie Zhao, Chengdong Peng, Shaofang Wang, Xinyue Liang, Xiaoyan Meng

**Affiliations:** 1 Department of Radiology, Tongji Hospital, Tongji Medical College, Huazhong University of Science and Technology, Wuhan, 430030 Hubei China; 2 United Imaging Healthcare, Shanghai, China

**Keywords:** Kidney, Magnetic resonance imaging, ACS, T2-weighted imaging

## Abstract

**Object:**

To explore the feasibility and clinical application of AI -assisted compressed sensing (ACS) technology in kidney MR imaging.

**Methods:**

33 patients were enrolled in this study, affiliated to our hospital from September 2020 to April 2021. The patients underwent T2-weighed sequences of both the ACS scan and the conventional respiratory navigator (NAVI) scan. We evaluated the subjective image quality scores, including the sharpness of image edge, artifact and the overall image quality, and compared the objective image quality indicators such as scanning time, signal-to-noise ratio (SNR), and contrast signal-to-noise ratio (CNR). The Wilcoxon’s rank sum test and the paired t test were used to compare the image quality between ACS and NAVI groups. The *p*-value less than 0.05 indicated a statistically significant difference.

**Results:**

The edge sharpness of the ACS group was significant lower than that of the NAVI group (*p* < 0.01), however, there were no significant differences in the artifact and the overall rating of image quality between the two groups (*p* > 0.05). In terms of the objective image quality scores, the scanning time of the ACS group is significantly lower than that of control group. The SNR and CNR of ACS group were significantly higher than those of NAVI group (SNR:3.63 ± 0.76 vs 3.04 ± 0.44, *p* < 0.001; CNR: 14.44 ± 4.53 vs 12.05 ± 3.32, *p* < 0.001). In addition, the subjective and objective measurement results of the two radiologists were in good agreement (ICC = 0.61–0.88).

**Conclusion:**

ACS technology has obvious advantages when applied to kidney MR imaging, which can realize ultra-fast MR imaging. The images can be acquired with a single breath-hold (17 s), which greatly shortens the scanning time. Moreover, the image quality is equal to or better than the conventional technology, which can meet the diagnostic requirements. Thus, it has obvious advantages in diagnosis for kidney disease patients with different tolerance levels for the clinical promotion.

**Supplementary Information:**

The online version contains supplementary material available at 10.1186/s12880-022-00842-1.

## Introduction

The kidney plays an important role in maintaining the human body’s environment, its main function is to form urine and excrete metabolites, and maintain the body's water, electrolyte and acid–base balance. MRI can realize multi-parameter and multi-directional imaging of kidney, which has important application value in kidney disease diagnosis and prognosis monitoring [1], but long exam time is one of its drawbacks, which makes it difficult for some patients to cooperate to complete the inspection, and it is prone to cause artifacts due to obvious motion artifacts or uneven breathing caused by body motion [2]. In addition, the amounts of clinical examinations are increasing, and there is an urgent need for innovative accelerated imaging to achieve ultra-fast scanning while obtaining high-quality images [3]. To solve these problem, various methods were developed for imaging acceleration, including compressed sensing (CS), Parallel imaging and Half Fourier (HF) acquisition [3–6]. The first two methods are based on linear mathematical models, the image quality at high acceleration factors can be reduced due to noise amplification [7, 8]. Compressed sensing uses a nonlinear mathematical model, which can effectively suppress the noise bands and artifacts caused by acceleration [9]. These methods effectively shorten the imaging time, while undoubtedly reducing the image quality.

To overcome the drawbacks of the acceleration techniques mentioned above, AI-assisted Compressed Sensing (ACS) technology is proposed to provide an integral MR acceleration solution, which combines CS, PI, HF and AI [10–12]. In ACS, an AI module based on deep learning neural network is innovatively introduced, which was trained with millions of fully-sampled and corresponding under-sampled data as ground truth and input to suppress artifacts and noise, with AI module collaborating with Half Fourier, Parallel Imaging and Compressed Sensing for noise suppression, artifact reduction and information recovery. With the ability of maintaining optimal image quality at higher acceleration factor, ACS can improve image quality without prolonging imaging time. It can realize each body part examination within 100 s, which is closer to the scanning speed of CT.

In this study, we aim to apply the ACS technology in kidney scans to achieve high-resolution T2 imaging of the kidney within an ultra-short time. We compare the subjective and objective image quality of the conventional navigator technology (NAVI) and the ACS to confirm the feasibility of ACS in kidney.

## Material and methods

### Patient information

This prospective study was performed on the patients for abdominal MR imaging in our hospital from September 2020 to April 2021, who underwent both ACS sequence scan and conventional T2 NAVI sequence scan. Exclusion criteria: (1) patients with contraindications in MRI examination; (2) patients who cannot hold their breath; (3) those whose image quality cannot meet the diagnostic requirements. There were 17 male patients and 16 female patients, with an average age of 50.4 ± 16.4 years, and an average glomerular filtration rate of 95.4. The basic information of the enrolled patients was shown in Table 1.

**Table 1:** Patients’ characteristics

	Patient information
Number of patients	33
Number of male patients	17
Number of female patients	16
Age (years old)	25–83
Mean age (years old)	50.1
EGFR, mL/min	95.4 (66.3–131.3)

### Inspection methods

All images were obtained in the supine position by using 12-channel abdominal phase array coil and spine matrix coil with a 3.0 T MRI scanner. Before scanning, the operating technician trained the patients with breath-hold to ensure that the patient can cooperate the inspection. The patient undergoes the conventional T2 NAVI sequence scan first, and then undergoes the ACS sequence scan. The scan parameters of the two sequences are detailed in the Table 2 below.

**Table 2:** Scan parameters list

Parameters	ACS	T2 NAVI
TR (ms)	10,785	4000
TE (ms)	95.76	136
Slice (mm)	5	5
Interlayer spacing (mm)	2	2
Scan time (s)	17	120–300
Flip angle (°)	90	110

### Objective image quality

All images were analyzed and processed on the workstation by the radiologists with more than 5 years of work experience. We measured the signal intensity of the kidney parenchyma and erector spinae muscle on the same scan level. The ROIs were about 90 mm^2^ and 110 mm^2^, respectively, avoiding the surrounding blood vessels and adipose tissue. Three regions of interest are drawn on the same level, and their average values were used as the tissue signal intensity to calculate the signal to noise ratio (SNR) and contrast to noise ratio (CNR). The calculation formulas were as follows:

$$SNR = \frac{{{\text{SI}}\;{\text{renal}}\;{\text{parenchyma}}}}{{SD\;{\text{renal}}\;{\text{parenchyma}}}}$$

$$CNR = \frac{{{\text{SI}}\;{\text{renal}}\;{\text{parenchyma}} - {\text{SI}}\;{\text{erector}}\;{\text{spinae}}\;{\text{muscle}}}}{{\sqrt {SD\;{\text{renal}}\;{\text{parenchyma}}^{2} + SD\;{\text{erector}}\;{\text{spinae}}\;{\text{muscle}}^{2} } }}$$

### Subjective image quality

Radiologists who have been working for 5 years scored the image quality subjectively. The scoring standards are as follows:

The sharpness of the image edge: 1 point = poor; 2 points = acceptable; 3 points = good; 4 points = very good.

artifacts: 1 point = excessive artifacts and cannot be diagnosed; 2 points = large artifacts and still diagnosable; 3 points = small artifacts; 4 points = no artifacts.

Overall image quality: 1 point = poor; 2 points = acceptable; 3 points = good; 4 points = very good.

### Statistical analysis

The SPSS software was used for statistical analysis of data. The interobserver agreement was assessed using the intraclass correlation coefficient (ICC) (0.00–0.20, poor agreement; 0.21–0.40, fair agreement; 0.41–0.60; moderate agreement; 0.61–0.80, good agreement; greater than 0.81, excellent agreement). The subjective scores of the two groups of images were statistically analyzed by Wilcoxon’s rank sum test. The objective scores of the images between the two groups were statistically analyzed by the paired t test.

## Results

### Kidney imaging obtained by conventional T2 NAVI and ACS technology

**Fig. 1:**
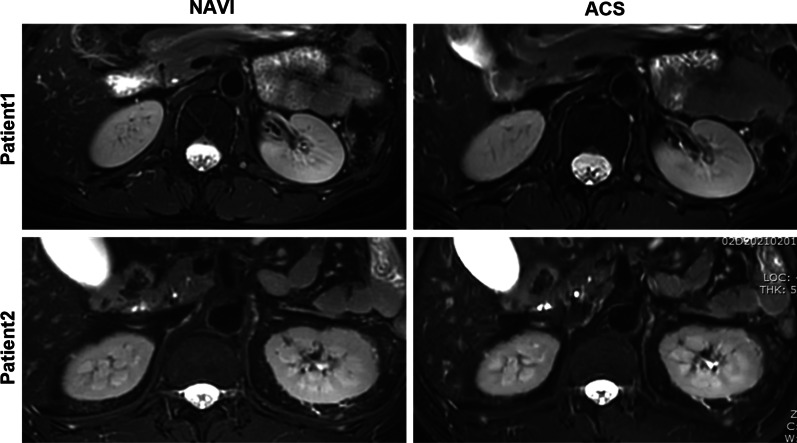
The kidney imaging obtained by ACS and T2 NAVI technique

These 33 patients enrolled in the group underwent both conventional T2 NAVI scanning and ACS scanning; compared with the T2 NAVI sequence, the scanning time of ACS technology was significantly shortened, the images could be acquired within17 seconds. The signals of the T2 NAVI sequence were collected by means of diaphragmatic navigation with free breathing. Its scanning time varies according to the respiratory rate, it usually takes about 3–5 min. Thus, the application of ACS technology not only shortens the acquisition time, but also obtains high-resolution kidney tissue information, and its image quality is equivalent to the conventional T2 NAVI technology level (as shown in Fig. 1).

### Subjective image quality

**Fig. 2:**
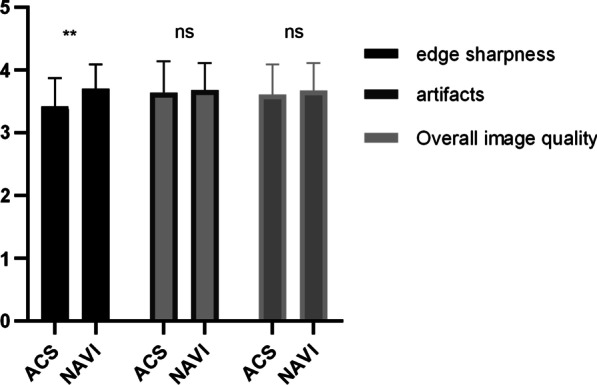
Subjective image quality rating scales of ACS and conventional groups

Two radiologists subjectively scored the edge sharpness, artifacts and overall image quality of these two groups. The image edge sharpness of the ACS group was lower than that of the NAVI group, but there was no significant difference in image artifacts and overall image scores between the two groups. The image edge sharpness, artifacts and overall image scores were: 3.42 ± 0.45 vs. 3.70 ± 0.39, *p* = 0.004; 3.64 ± 0.50 vs 3.68 ± 0.43 *p* = 0.56; 3.61 ± 0.48 vs 3.67 ± 0.44, *p* = 0.43 (ACS vs NAVI), which is shown in Fig. 2.

### Objective image quality

**Fig. 3:**
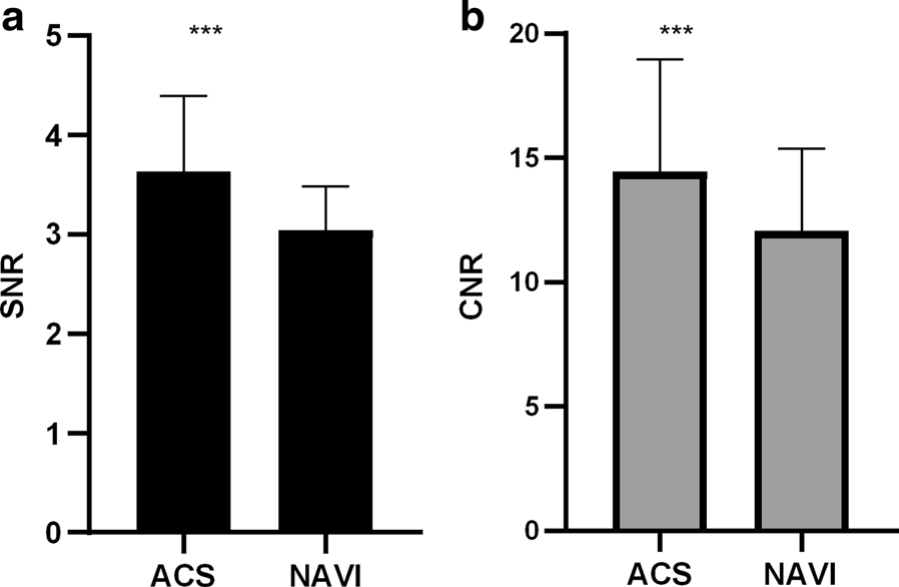
Objective image quality scores in ACS and T2 NAVI groups

The objective scoring items include SNR and CNR. Two radiologists measured the SNR and CNR of the two groups. There were differences in the SNR and CNR of the two groups of images. SNR and CNR were 3.63 ± 0.76 vs 3.04 ± 0.44, *p* = 0.0004; 14.44 ± 4.53 vs 12.05 ± 3.32 *p* = 0.0004 (ACS vs NAVI), as shown in Fig. 3. The SNR and CNR of the ACS group are both higher than that of NAVI group.

### ICC consistency score

The images were scored and measured by two radiologists, and each radiologist used the same evaluation criteria and measurement methods. During the measurement process, due to the difference in the location of the ROI on the kidney, the final result may be affected, so two radiologists carried out the consistency analysis of measurement data. As shown in Table 3, the subjective and objective scores of the two physicians are in good agreement. (ICC 0.00–0.20, poor agreement; 0.21–0.40, fair agreement; 0.41–0.60; moderate agreement; 0.61–0.80, good agreement; greater than 0.81, excellent agreement).

**Table 3:** ICC consistency score

	ACS			NAVI		
	R1	R2	ICC	R1	R2	ICC
Image sharpness	3.39 ± 0.50	3.45 ± 0.51	0.78	3.70 ± 0.47	3.45 ± 0.51	0.61
Image artifacts	3.70 ± 0.53	3.56 ± 0.56	0.83	3.76 ± 0.44	3.61 ± 0.56	0.64
Overall image quality	3.61 ± 0.50	3.61 ± 0.45	0.93	3.73 ± 0.45	3.61 ± 0.50	0.85
CNR	15.4 ± 5.20	13.48 ± 4.87	0.73	12.05 ± 3.98	11.6 ± 3.53	0.71
SNR	3.61 ± 0.79	3.66 ± 0.82	0.88	3.05 ± 0.47	3.02 ± 0.50	0.76

## Discussion

In this study, we explored the feasibility and preliminary investigation of ACS technology in kidney MR imaging. We compared the T2WI serial axial images obtained by traditional NAVI and the new ACS technology, the items contain subjective and objective image quality scores to discuss the value of ACS technology.

The subjective image quality scoring items include image sharpness, artifacts and overall image quality. The results showed that the edge sharpness of the ACS group was lower than that of the conventional group, but there was no significant difference between the two groups of images in image artifacts and overall image quality. The images in the ACS group were obtained in a single breath-hold. While the images in the NAVI group were obtained by diaphragmatic navigation with free breathing. Theoretically, the image quality of one breath-hold is better than that of the breath-triggered image [13]. If the patient’s breathing is very regular, the image by breath triggering or diaphragmatic navigation may be better. The advantage of breath-hold is in faster scanning and better stability. However, due to the limitation of breath-hold time, the echo chain is longer. At the same time, the introduce of some filter parameters produces fuzzy artifacts to a certain extent. Therefore, the image sharpness of the ACS group is lower than that of the conventional NAVI group. Besides, image quality is affected by many factors, such as parameter settings, patient's breathing rate, etc. Thus, there is no significant difference between the two groups in image artifacts and overall image quality.

The objective image quality scoring items include scan time, SNR and CNR. For kidney tissues, T2-weighted sequence can provide kidney tissue morphology and pathological features, which is obtained by respiratory triggering/diaphragmatic navigation or breath hold. The images of NAVI group are acquired in 3–5 min with free breathing. A major problem with NAVI scans is that the acquisition times are unpredictable and vary widely. By contrast, the images in ACS group are acquired in a single breath hold, it takes about 17 s to capture images. And the reconstruction times of the ACS technique is very soon, the reconstructed images appear at the end of the scanning. Comped with the conventional T2 NAVI technology, ACS can significantly shorten the scan time, realize ultra-fast scan of the kidney, and increase the scan time by 10–17 times. When some patients with severe diseases undergo imaging examinations, it is often difficult to maintain a static state for a long time due to pain, disturbance of consciousness and other reasons, resulting in artifacts and reducing image quality. ACS technology can not only solve this problem, but also improve image quality and diagnostic accuracy. Moreover, the SNR and CNR of the ACS group were higher than those of the conventional NAVI group (Fig. 3), which is consistent with theory. The image obtained in a breath-hold will be better than the image quality triggered by free breathing. In summary, the ACS technology can not only effectively shorten the scanning time, but the objective image quality is also higher than that of the conventional group.

Due to the subjective differences of physicians such as the placement of the ROI, the results may make a difference. Therefore, we conducted a consistency analysis of the measurement results of the two physicians to evaluate the credibility of the data. The results showed that the subjective image quality and objective image quality scores are in good or excellent agreement, and they are expected to be widely used in clinical practice.

However, this study also has some shortcomings. The sample size is not large enough, which may cause bias in data selection. Secondly, missing comparison with alternative techniques is the limitation. In this article we compared the ultra-fast T2WI imaging technology (ACS) with the traditional group. In our hospital, the images of the traditional group are obtained by diaphragmatic/respiratory navigation with free breathing. Thus, we only compared the ACS group with NAVI group. The Additional file 1 contains the comparison with alternative techniques. At the same time, this study did not include the patients with kidney disease. In future studies, we plan to include patients with renal insufficiency and renal space occupation for research to better verify the possibility of clinical application of ACS technique.

In summary, ACS technique can realize ultra-fast MR imaging of kidney with higher subjective and objective image quality with consistent and reliable acquisitions. It is worth pointing out that the scanning time is 17 s, which is more suitable for patients with different tolerance levels, and the work efficiency of radiology technicians can be significantly improved. Therefore, this ultra-fast scanning method is worthy of clinical widespread promotion.

## Supplementary Information

**Additional file 1**: **Table S1**. The raw data of objective image quality. **Table S2**. The raw data of subjective image quality by Radiologist 1 (R1). **Table S3**. The raw data of subjective image quality by Radiologist 2 (R1). **Table S4**. The raw data of subjective image quality (mean value of the scoring). **Table S5**. The calculated data about objective/subjective image quality. **Table S6**. Subjective image quality rating scales and Objective image quality scores in ACS and SSFSE groups. **Fig. S1**. The typical kidney images obtained by ACS and SSFSE technique. **Fig. S2**. The histogram about subjective image quality rating scales and objective image quality scores in ACS and SSFSE groups.

## Data Availability

The authors declare that all data supporting the fundings of this study are available within the paper and its source data for the figures and tables in this study are available from the corresponding authors upon request.

## Abbreviations

ACS: AI-assisted compressed sensing
MRI: Magnetic resonance imaging
SNR: Signal to noise ratio
CNR: Contrast to noise ratio
ICC: Intraclass correlation coefficient
