# Supplementary material for: The feasibility investigation of AI -assisted compressed sensing in kidney MR imaging: an ultra-fast T2WI imaging technology
Source: BMC Med Imaging. 2022 Jul 4;22:119. doi: 10.1186/s12880-022-00842-1 (PMC9254529; doi:10.1186/s12880-022-00842-1)
Supplement: Supplementary file 1 — Additional file 1: Table S1. The raw data of objective image quality. Table S2. The raw data of subjective image quality by Radiologist 1 (R1). Table S3. The raw data of subjective image quality by Radiologist 2 (R1). Table S4. The raw data of subjective image quality (mean value of the scoring). Table S5. The calculated data about objective/subjective image quality. Table S6. Subjective image quality rating scales and Objective image quality scores in ACS and SSFSE groups. Fig. S1. The typical kidney images obtained by ACS and SSFSE technique. Fig. S2. The histogram about subjective image quality rating scales and objective image quality scores in ACS and SSFSE groups. [file 12880_2022_842_MOESM1_ESM.docx]

**The feasibility investigation of** **AI -assisted Compressed Sensing in kidney MR imaging: An ultra-fast T2WI imaging technology**

Yanjie Zhao^1^, Chengdong Peng^1^, Shaofang Wang^1^, Xinyue Liang^2^, Xiaoyan Meng^1*^

^1^ Department of Radiology, Tongji Hospital, Tongji Medical College, Huazhong University of Science and Technology, Wuhan, Hubei, 430030

^2^ United Imaging Healthcare, Shanghai, China,

**Supplementary Tables of Raw Data**

1. The raw data of objective image quality is shown below (Supplementary Table 1), two radiologists measured the SNR and CNR of the two groups (ACS group vs. NAVI group). R1 stands for radiologist 1. R2 stands for radiologist 2.

**Supplementary Table 1.** The raw data of objective image quality.


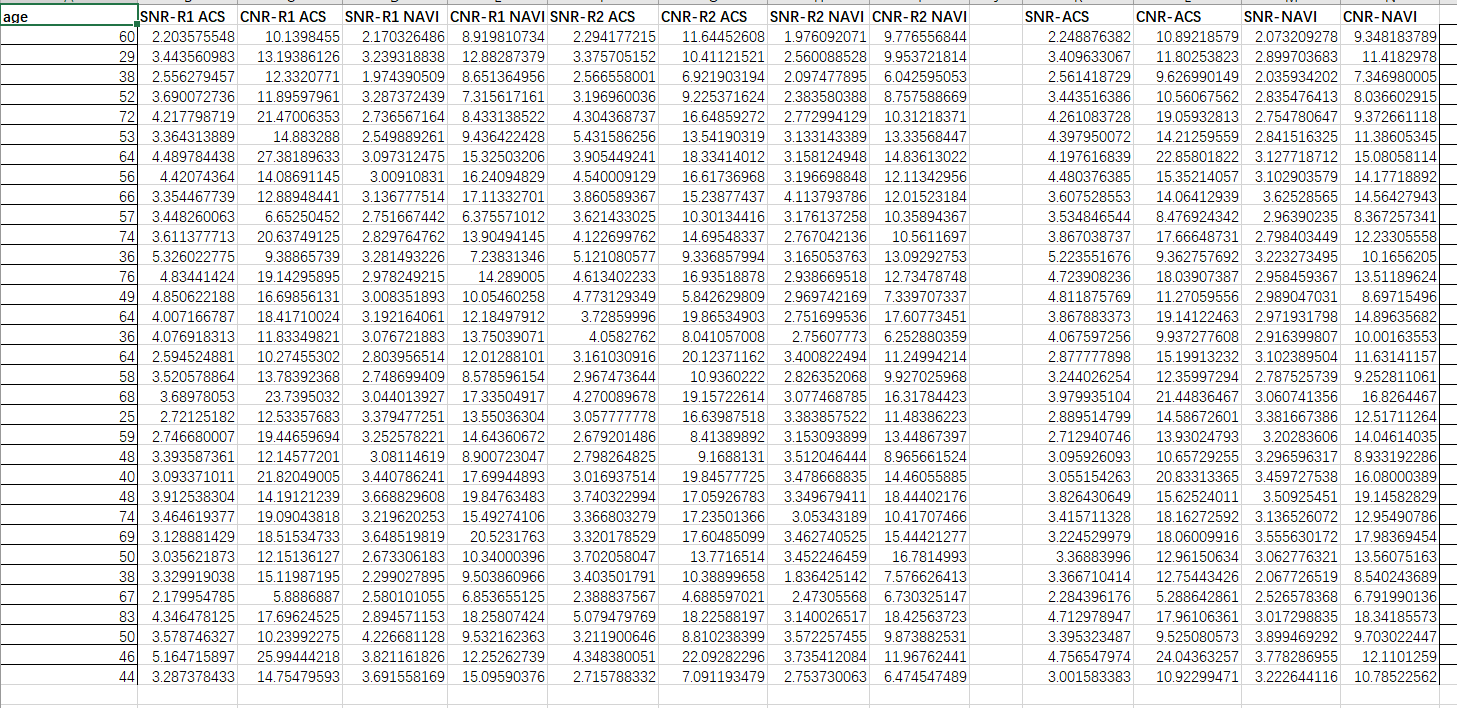


1. The raw data of subjective image quality is shown below, two radiologists subjectively scored the edge sharpness, artifact and overall image quality of the two groups (ACS group vs. NAVI group). Supplementary Table 2 and Supplementary Table 3 show the scoring of Radiologist 1 and Radiologist 2, respectively. Supplementary Table 4 show the mean value of scoring by the two Radiologists.

**Supplementary Table 2.** The raw data of subjective image quality by Radiologist 1 (R1).


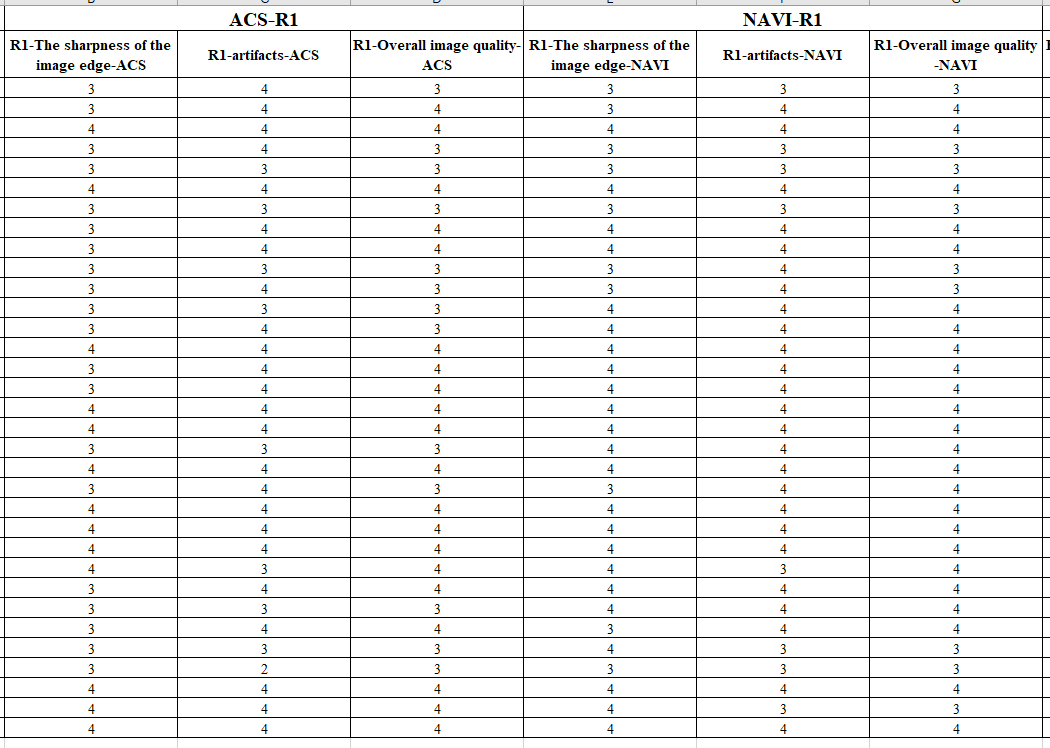


**Supplementary Table 3**. The raw data of subjective image quality by Radiologist 2 (R1).


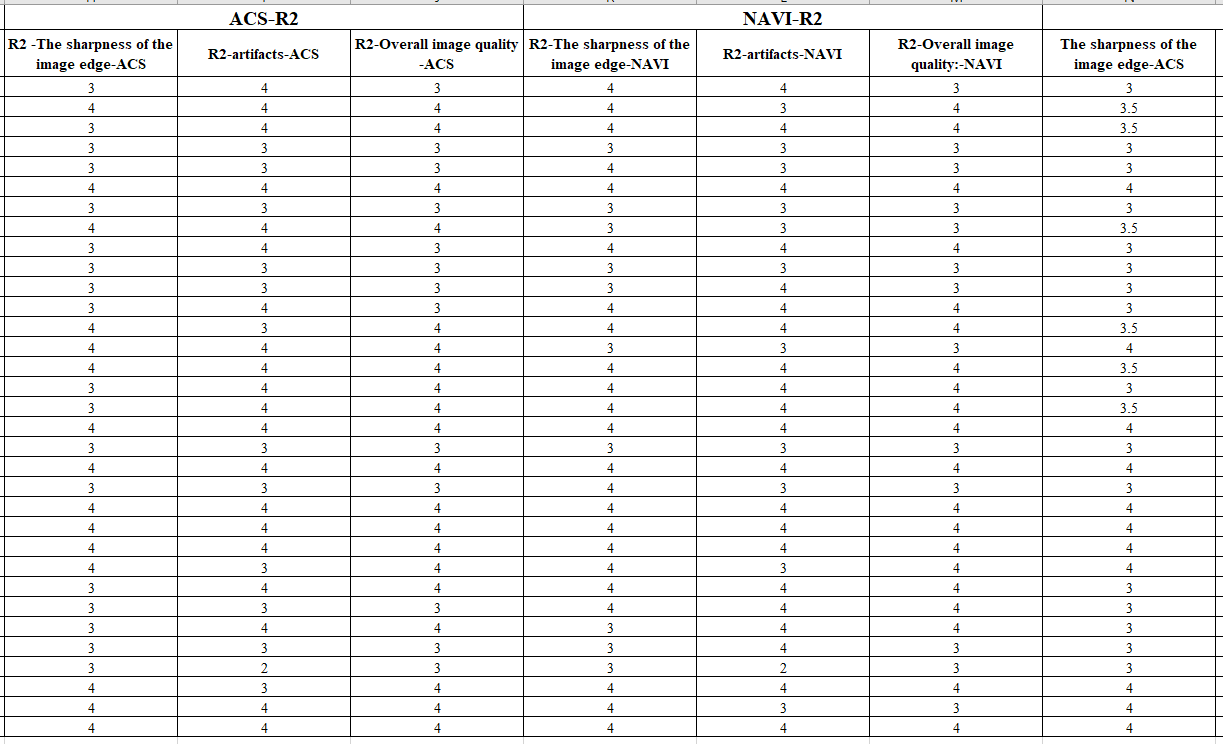


**Supplementary Table 4**. The raw data of subjective image quality (mean value of the scoring).


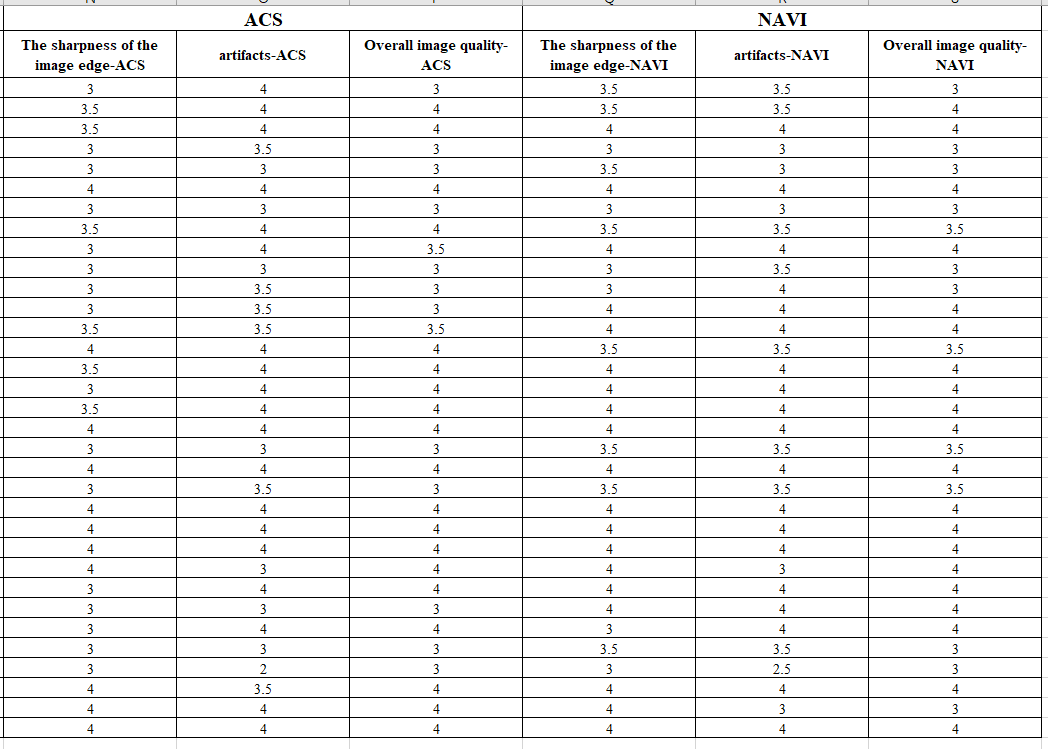


1. The calculated data about objective/subjective image quality is shown as below (Supplementary Table 5).

**Supplementary Table 5**. The calculated data about objective/subjective image quality.

|  | **ACS** | | **NAVI** | | | |
| --- | --- | --- | --- | --- | --- | --- |
|  | **R1** | **R2** | **R1** | **R2** | |  |
| **Image sharpness** | 3.39±0.50 | 3.45±0.51 | 3.70±0.47 | | 3.45±0.51 | |
| **Image artifacts** | 3.70±0.53 | 3.56±0.56 | 3.76±0.44 | | 3.61±0.56 | |
| **Overall image quality** | 3.61±0.50 | 3.61±0.45 | 3.73±0.45 | | 3.61±0.50 | |
| **CNR** | 15.4±5.20 | 13.48±4.87 | 12.05±3.98 | | 11.6±3.53 | |
| **SNR** | 3.61±0.79 | 3.66±0.82 | 3.05±0.47 | | 3.02±0.50 | |

**Supplementary data in ACS and SSFSE group**

1) We compared the ACS group (ACS breath-holding state) with new group (SSFSE breath-holding state). Supplementary Figure 1 show the typical kidney images obtained by ACS and SSFSE technique. The images of ACS group are obtained within 17 s (single-breath hold), the images of SSFSE group are obtained within 31 s (two-breath hold).


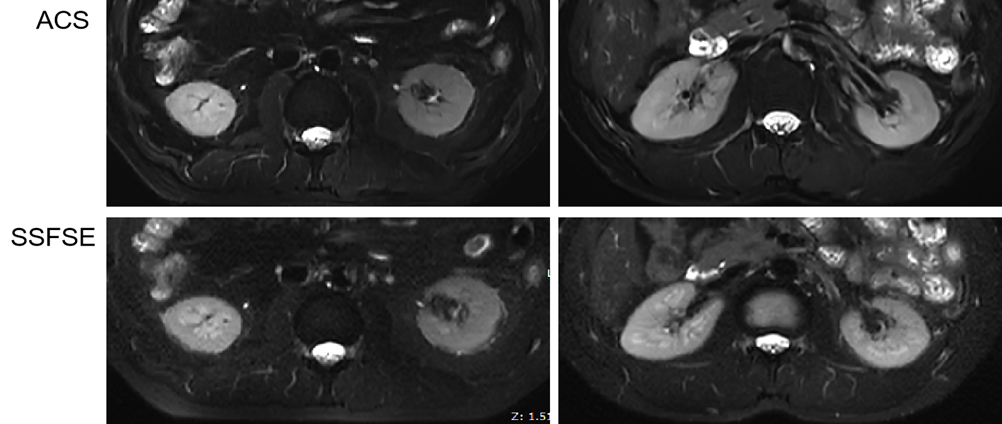


**Supplementary Figure 1**. The typical kidney images obtained by ACS and SSFSE technique.

2) The subjective image quality in ACS group is higher than that in SSFSE group. The SNR of the ACS group is higher than that of the SSFSE group, and the CNR of the ACS group is lower than that of the SSFSE group. Which is shown in Supplementary Table 6 and Supplementary Figure 2.

**Supplementary Table 6.** Subjective image quality rating scales and Objective image quality scores in ACS and SSFSE groups.

|  | **ACS** | **SSFSE** | **P** |
| --- | --- | --- | --- |
| **sharpness** | 3.77±0.43 | 2.59±2.50 | ＜0.001 |
| **artifact** | 3.95±0.21 | 2.86±0.35 | ＜0.001 |
| **overall score** | 3.86±0.35 | 2.82±0.39 | ＜0.001 |
| **CNR** | 5.25±1.07 | 5.69±1.20 | =0.005 |
| **SNR** | 19.58±6.23 | 12.34±2.88 | ＜0.001 |

**
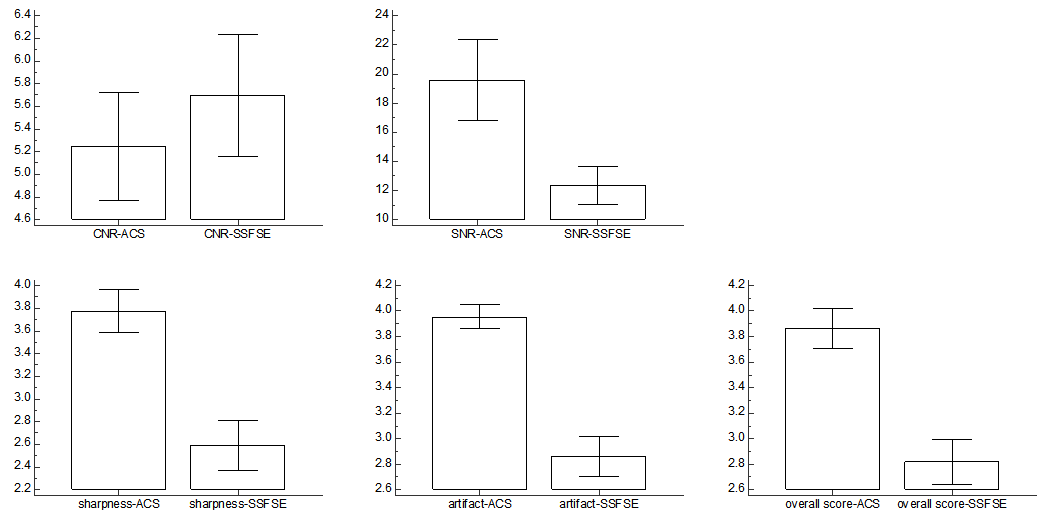
**

**Supplementary Figure 2.** The histogram about subjective image quality rating scales and objective image quality scores in ACS and SSFSE groups.
